# Automatic deep learning-based pleural effusion segmentation in lung ultrasound images

**DOI:** 10.1186/s12911-023-02362-6

**Published:** 2023-11-29

**Authors:** Damjan Vukovic, Andrew Wang, Maria Antico, Marian Steffens, Igor Ruvinov, Ruud JG van Sloun, David Canty, Alistair Royse, Colin Royse, Kavi Haji, Jason Dowling, Girija Chetty, Davide Fontanarosa

**Affiliations:** 1 School of Clinical Sciences, Queensland University of Technology, Gardens Point Campus, 2 George St, Brisbane, QLD 4000 Australia; 2 Centre for Biomedical Technologies (CBT), Queensland University of Technology, Brisbane, QLD 4000 Australia; 3 Department of Surgery (Royal Melbourne Hospital), University of Melbourne, Royal Parade, Parkville, VIC 3050 Australia; 4 CSIRO Health and Biosecurity, The Australian eHealth Research Centre, Herston, QLD 4029 Australia; 5 Department of Electrical Engineering, Eindhoven University of Technology, 5600 MB Eindhoven, The Netherlands; 6 Department of Medicine and Nursing, Monash University, Wellington Road, Clayton, 3800 Victoria Australia; 7 Outcomes Research Consortium, Cleveland Clinic, Cleveland, Ohio USA; 8 School of IT & Systems, Faculty of Science and Technology, University of Canberra, 11 Kirinari Street, Bruce, ACT 2617 Australia

**Keywords:** Point-of-Care Ultrasound, Lung Ultrasound, Deep Learning, Pleural Effusion / diagnostic imaging

## Abstract

**Background:**

Point-of-care lung ultrasound (LUS) allows real-time patient scanning to help diagnose pleural effusion (PE) and plan further investigation and treatment. LUS typically requires training and experience from the clinician to accurately interpret the images. To address this limitation, we previously demonstrated a deep-learning model capable of detecting the presence of PE on LUS at an accuracy greater than 90%, when compared to an experienced LUS operator.

**Methods:**

This follow-up study aimed to develop a deep-learning model to provide segmentations for PE in LUS. Three thousand and forty-one LUS images from twenty-four patients diagnosed with PE were selected for this study. Two LUS experts provided the ground truth for training by reviewing and segmenting the images. The algorithm was then trained using ten-fold cross-validation. Once training was completed, the algorithm segmented a separate subset of patients.

**Results:**

Comparing the segmentations, we demonstrated an average Dice Similarity Coefficient (DSC) of 0.70 between the algorithm and experts. In contrast, an average DSC of 0.61 was observed between the experts.

**Conclusion:**

In summary, we showed that the trained algorithm achieved a comparable average DSC at PE segmentation. This represents a promising step toward developing a computational tool for accurately augmenting PE diagnosis and treatment.

## Introduction

Pleural effusion (PE), the excessive accumulation of fluid in the pleural cavity, is the most common disease among all pleural diseases [1]. The most cited population study (Czech Republic) measured an incidence of 0.32 percent annually for PE [2]. In patients with COVID-19, the overall incidence of PE was 7.3 percent [3]. While history taking, a physical examination remains the basis for evaluating patients with PE, the British Thoracic Society in its latest draft guideline on pleural disease [4] has included a recommendation on performing lung ultrasound (LUS) on every patient’s initial presentation and when procedures involving the pleura are being performed. LUS provides a higher accuracy than physical examination [5] and chest radiography in the detection of PE [6]. However, LUS assessment typically involves a highly trained and experienced operator acquiring the images using a well-outlined protocol [7] and interpreting the images for pattern changes associated with lung pathology. The conventional LUS assessment, therefore, requires considerable time and resources [8].

Beyond recognizing and identifying PE, the underlying causes of PE are numerous, and an invasive procedure known as thoracentesis is frequently required to guide differential diagnosis and treatment [4]. The LUS-guided intervention has been shown to increase success at obtaining pleural fluid and, in turn, reduce the risk of complications such as pneumothorax [9]. This procedure is rapidly becoming standard patient care. To perform thoracentesis, recognition of the extent and location of the PE is critical, as a needle or catheter is introduced to the pleural space. A previous study from our group [10] implemented a deep learning (DL) based algorithm to detect the presence of PE on LUS. We demonstrated a comparable accuracy between the algorithm and the LUS experts [10]. This was the first step in supporting clinicians to identify image patterns associated with PE. Recognizing that LUS currently demands significant resources, and that LUS is highly desirable in patients with PE, we propose a DL-based algorithm to automatically segment PE to assist clinician(s) in localizing PE in LUS videos for safe needle guidance during thoracentesis procedures, improve PE localization accuracy, and aid clinicians during PE volume estimation. This automated algorithm for segmenting PE could provide considerable benefits to initial differential diagnosis, subsequent patient safety, and improved procedural success if a more invasive diagnostic and treatment approach is warranted. The performance of the proposed algorithm was evaluated using the Dice Similarity Coefficient (DSC) and the complementary Szymkiewicz-Simpson Overlap Similarity Coefficient (OVC) against the LUS experts. The interobserver variability between the LUS experts’ contours and the algorithms’ performance on PE segmentations were evaluated using the DSC and OVC metrics.

This study’s central aim is to confront the current hurdles in developing an automated segmentation method for PE detection, a previously unexplored territory. Additionally, it builds upon prior work [11] by delving into the assessment of segmentation variations among experts. Existing research on LUS PE segmentation is limited, with only one study available, which does not perform segmentation on a per-patient basis where the frames are not divided into the training, validation and test set per patient. Additionally, this study relies solely on average Dice scores for the entire dataset and lacks an evaluation metric that assesses the common overlapped area between segmentations, such as the Szymkiewicz-Simpson overlap coefficient.

This study aims to address these gaps by establishing a comprehensive LUS scanning protocol that covers various potential lung pathologies, with a particular focus on addressing PE as an initial step. The deep learning approach builds on previous classification work and addresses the need for automated segmentation of imaging patterns and artifacts in the lung that indicate PE. This segmentation enables the visual presentation of PE regions to sonographers, facilitating the diagnostic process of fluid drainage in the lung (thoracentesis). Furthermore, it lays the foundation for potential sonographer image guidance in future applications and research.

### Related works

The ability to segment LUS images has been explored for several respiratory pathologies. Most notably, Morilhat et al. [12] recently shared their findings from a DL-based algorithm (nnU-net) for PE segmentation with median DSC scores of 0.74 and 0.82 for two datasets consisting of patients from low-to-middle-income countries suspected of tuberculosis. The group performed an automatic DL-based segmentation using spatial information consisting of 2D pixel coordinate information as an additional input into their algorithm. Their algorithm performance was evaluated on a per-dataset basis and compared to the experts’ through an interobserver study. Our study differs from Morilhat et al. [12] in several aspects, including a different patient cohort based on an ultrasound (US) imaging acquisition protocol that focused on LUS pathology identification, DSC/OVC evaluations done on a per patient basis using an improved clinical contouring criteria, US probe settings, and a CNN (with Reg-STN) based algorithm.

The other studied LUS image patterns included COVID-19 markers [13–15] for producing an automated COVID-19 associated pathologies segmentation tool. Roy et al. [14] implemented a weakly supervised classification algorithm using a Spatial Transform Network (STN) and a segmentation algorithm to identify and spatially localize pathology image patterns associated with COVID-19 markers in LUS videos, respectively. In addition, Mento et al. [13] used the same algorithm on new sets of COVID-19 data and applied a threshold technique to the experts’ and algorithm’s frame segmentations to improve the video-based agreement between the algorithm predictions and the experts’ ground truths. In contrast, Roshan et al. [15] performed automatic segmentation of COVID-19 associated imaging patterns using a modified U-net structure and included the OVC to further determine the agreement between the predicted and the ground truth segmentations.

## Materials and methods

This study was approved by The Melbourne Health Human Research Ethics Committee (Australia) (28/08/2018, ACTRN12618001442291, HREC/66935/MH-2020) and was performed in accordance with the Declaration of Helsinki. LUS images used in this study were acquired from a previous study [16]. Written informed consent was obtained from all participating patients [16] (Melbourne Health Human Research Ethics Committee approval HREC/18/MH/269, trial registration: http://www.ANZCTR.org.au/ACTRN12618001442291.aspx).

**Fig. 1:**
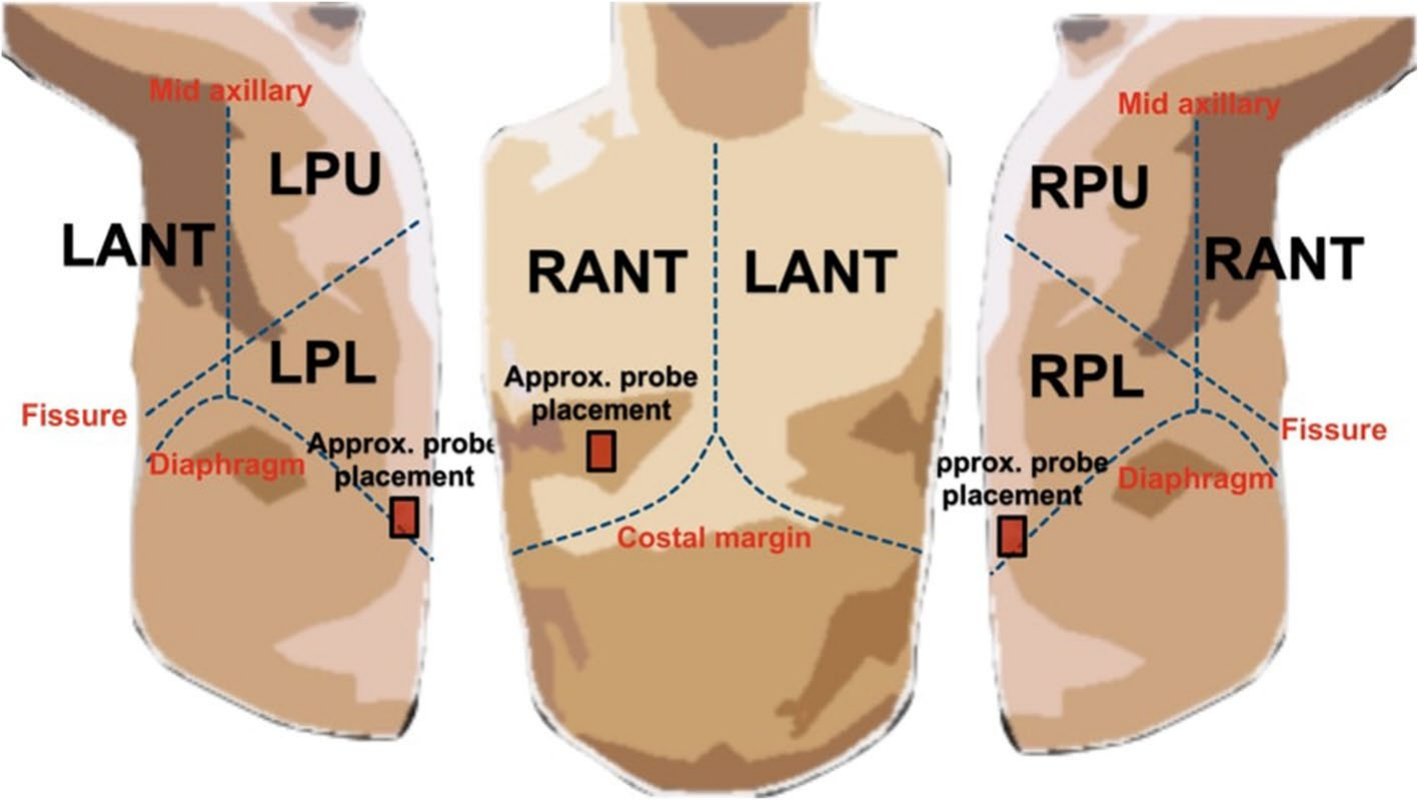
Examples of the scanning regions (viz. Right Anterior (RANT), Right Posterior Lower (RPL), Left Posterior Lower (LPL)) and the approximate probe placement during the image acquisition of LUS frames containing PE. $$^{**}$$Figure created and owned by coauthors

All patients were admitted to the Royal Melbourne Hospital under an Internal Medicine unit with a cardiorespiratory-related presentation. The LUS examination was performed by an experienced physician trained (XC) in point-of-care US (POCUS) [16] using a Sonosite X-Porte portable US imaging system (Fujifilm, Bothell, WA, USA) with settings shown in Table 1 [17]. The examination followed a standardized iLungScan protocol (The University of Melbourne, Ultrasound Education Group [17]). Patients were positioned in a supine position, and six distinct lung scanning zones [18] were examined at least once (Fig. 1). Images were immediately reviewed for diagnostic accuracy and quality assurance by a second LUS expert (DC, AR, or CR).

A total of 51 LUS videos from 24 patients were used to train our automated PE segmentation algorithm. The algorithm was trained on approximately an equal number of expert 1 and expert 2 ground truths during hyperparameter optimization. The cross-validation was performed on a per patient level using each expert’s segmentation labels and calculating the DSC/OVC scores between the experts and the algorithm-predicted segmentation.

**Table 1:** Ultrasound imaging system parameters [17]

Sonosite X-Porte	
Probe/Transducer	Phased Array
Preset	Cardiac
Frequency	1-5 MHz
Acoustic working Frequency	1.72 MHz
Mechanical index	1.3
Soft-tissue thermal index	0.9
Focal optimization	Gen
Default penetration depth	15 cm
Pulse Repetition	2933 Hz
Scan repetition rate	34.1 Hz
Tissue harmonic imaging	On

### Dataset

**Fig. 2:**
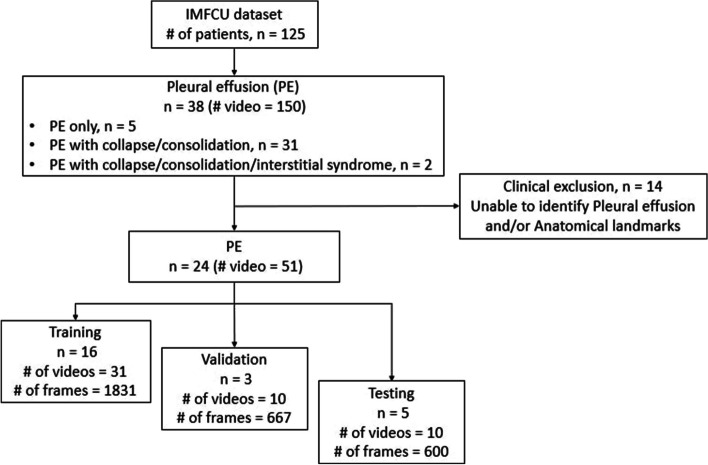
A flow diagram showing the pathology distribution of the original PE patients to the final training/validation/test dataset split used to train the algorithm

The 38 PE patient dataset (detailed in Fig. 2) was obtained based on a six-scanning region LUS image acquisition protocol [17, 18] shown in Fig. 1. These six scanning regions include the Left Anterior (LANT), Right Anterior (RANT), Left Posterior Upper (LPU), Left Posterior Lower (LPL), Right Posterior Upper (RPU), and Right Posterior Lower (RPL) regions.

The dataset from Table 2 has been further analyzed, according to the clinical criteria consisting of the experts’ (trained sonographer (MS) and trained LUS MD (AW)) ability to identify PE imaging patterns, which is further detailed in “Frame-based contouring strategy” section. As a result, the number of PE patients was reduced from 38 to 24 as shown in Fig. 2. Table 2 outlines the number of patients, videos and frames for each of the pathologies that presented alongside PE and is the dataset that is used to train and test our algorithm.

**Table 2:** Breakdown of PE patients during algorithm training/testing

	# Patients	# Videos	# Frames
**Effusion**	3	4	240
**Collapse / Consolidation / Effusion**	20	43	2561
**Acute Pulmonary Oedema / Interstitial Syndrome / Effusion**	1	4	240
**Total**	24	51	3041

**Table 3:** The patients from Table 2 and their respective scanning region distributions shown per video and per frame

	RANT	RPL	LPL
**Effusion**	N/A	# Video(s) 3 # Frame(s) 180	# Video(s) 1 # Frame(s) 60
**Collapse / Consolidation / Effusion**	# Video(s) 1 # Frame(s) 60	# Video(s) 15 # Frame(s) 900	# Video(s) 27 # Frame(s) 1601
**Acute Pulmonary Oedema / Interstitial Syndrome / Effusion**	N/A	# Video(s) 1 # Frame(s) 60	# Video(s) 3 # Frame(s) 180

Table 3 shows the scanning regions and the number of videos/frames associated with the patient pathology distribution from Table 2. The PE dataset breakdown from Tables 2 and 3 show that PE patients present with multiple other pathologies (Consolidation/Collapse, APO, Interstitial Syndrome) other than PE. A significant representation of the dataset contains patients with a combined diagnosis of PE/Consolidation/Collapse located in the RPL and LPL LUS scanning regions.

### Pre-processing

The anonymization and removal of unique patient identifiers on the LUS videos with imaging patterns associated with PE was done before any segmentation labels could be completed. The next step involved extracting the LUS videos from the compressed Digital Imaging and Communications in Medicine [19] (DICOM) image format using Pydicom [20] and removing overlays (e.g., text, watermarks, trademarks, etc.) outside the US sector. The image pixel dimensions were $(0.02 \cdot 0.02) mm$. The original image size $(960 \cdot 720) pixels$ was reduced to $(806 \cdot 550) pixels$ by cropping the images to enclose the US sector, thus minimizing the presence of black pixels surrounding the relevant image information.

### Clinical PE contouring methods

Following image pre-processing, two reviewers (AW and MS) trained in LUS were assigned LUS videos in DICOM format for independent image interpretation. The reviewers did not have knowledge of the extent of PE on a video or frame level before reviewing the videos. The reviewers used a modified version of the Labelme [21] program to view and outline the PE. Sixty consecutive frames were selected from the videos most representative of the pathology at the reviewers’ discretion. Polygons were created to outline the PE on a frame-by-frame basis. The coordinates of the polygons were recorded in the open standard file format of JavaScript Object Notation [22] (JSON).

Identification of PE on LUS followed the Lung Ultrasound Interpretation Score protocol developed at the University of Melbourne Ultrasound Education Group, which is based on the international evidence-based LUS recommendations from Volpicelli et al. [23].

**Fig. 3:**
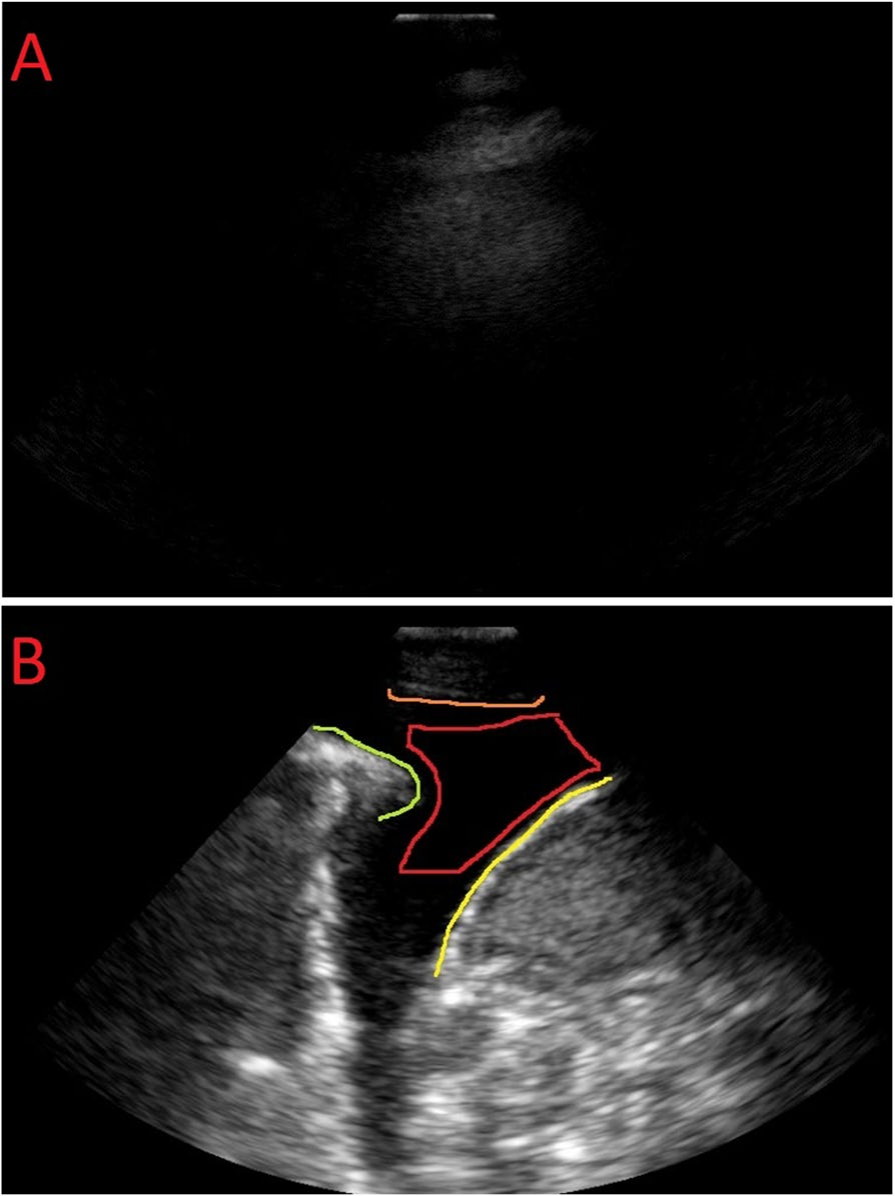
Comparison of PE identified LUS frames, where image (**A**) does not meet the clinical criteria needed for contouring PE and image (**B**) is the ideal case for PE segmentation. The presence of PE was defined as an anechoic space (in red) between the parietal pleura (lining the chest wall in orange and diaphragm in yellow) and visceral pleura (lining the lung surface in green) image B, along with the collapsed lung tissue (in green)

Figure 3 provides a visual representation of the exclusion criteria used in this study. In Image A, we can observe an LUS frame that is not suitable for training purposes due to several reasons, primarily centered around poor image quality. These issues include the absence of discernible anatomical or pathological markers. Image A represents one of the extreme cases of an inconclusive frame.

On the other hand, Image B in Fig. 3 illustrates an ideal LUS frame or video for PE identification. Here, the clinical significance lies in the clear visibility and identification of anatomical markers such as the collapsed lung, pleural lining, and diaphragm, along with the presence of PE-associated imaging patterns.

The LUS reviewers also outlined the PE using identifiable anatomical features, including the diaphragm, collapsed or consolidated lung, the chest wall, and the clear line that delineated the extent of the image sector. On completing one video frame, the reviewers repeated the same process on the subsequent frames. The same polygons could be transferred from the previous frame to the subsequent one, accounting for the pathology being continuous in the consecutive frames. However, the reviewers were required to examine each frame closely to ensure the polygon delineating PE on the previous frame remained applicable to the following frame. Depending on the amount of respirophasic movement of the lung and effusion, the reviewers had to adjust the polygons by applying the same diagnostic principle.

The contours for the final training and validation sets consisted 50% from expert 1 and 50% from expert 2. However, in the testing set, each patient had contours created by both experts.

### Frame-based contouring strategy

After application of the clinical data exclusion criteria, each LUS frame containing PE imaging patterns had an associated contour that was used as the ground truth segmentation label during training and assessment of the algorithm performance. These LUS contoured frames were then split into training, validation and testing sets based on the number of patients, and were divided into their respective videos, frames, and scanning regions as shown in Table 4. These training/testing dataset splits are based on the standard 80/20 split [24] to balance the variance between the training and testing performance.

**Table 4:** Distribution of PE patients following an 80/20 training/testing split that is further divided by the number of videos and frames used in the training, validation, and test sets

Set Type	# Patients	# Videos			# Frames		
Training	16	31			1831		
		RANT	RPL	LPL	RANT	RPL	LPL
		1	12	18	60	720	1051
Validation	3	10			610		
		RANT	RPL	LPL	RANT	RPL	LPL
		0	1	9	0	60	550
Testing	5	10			600		
		RANT	RPL	LPL	RANT	RPL	LPL
		0	6	4	0	360	240

### Deep learning model

The approach used leveraged a DL architecture that combined two key components: a Convolutional Neural Network (CNN) and a Spatial Transformer Network (STN) [14] into our framework to accurately pinpoint the presence of pulmonary pleural effusions (PE). Additionally, the CNN segment of our algorithm was built upon a customized U-net architecture.

This architecture allowed us to effectively identify and localize PE within lung ultrasound scans, contributing to the diagnostic process by automating the detection of this pathology. The STN played a crucial role in precisely mapping the regions of interest, while the CNN provided the necessary segmentation and classification capabilities for accurate detection.

The algorithm was trained by minimizing the pixel-wise categorical cross-entropy loss between the segmentation generated from the clinicians’ contours (ground truths) and the segmentations predicted by the algorithm [14]. Binary semantic segmentation was used, where one class label is used to represent the background of the LUS frame (pixels that have no PE segmentation), and the second class includes the segmentation that contains the image patterns associated with the PE pathology within a LUS frame.

### Training approach

The Weights & Biases [25] framework was utilised to perform hyperparameter tuning based on the validation set performance. The optimization of these hyperparameters was based on the Bayesian method [26], where the training was based on minimizing the training loss while taking into consideration the validation loss, validation DSC, and training DSC curves to prevent overfitting (Table 5).

**Table 5:** The hyperparameters used to train the PE segmentation model

Batch size	32
Batch normalization	True
Dropout	0.5
Epochs	50
Learning rate (Adam)	$10^{-5}$
Loss function	Binary Cross Entropy

The validation process plays a critical role in determining the optimal hyperparameters of the algorithm to ensure its best performance based on training from a specified dataset. This process ensures the robustness of these hyperparameters when the algorithm is subsequently tested on an independent set, which, in this study, consisted of 5 patients. Keeping consistency across the folds was crucial as it allowed for comprehensive testing of the algorithm’s performance, including evaluation against the interobserver study dataset and additional patients.

To achieve this, a repeated 5-fold cross-validation was conducted for each expert, resulting in a total of 10 folds. In each fold, one patient and their associated videos were replaced in the original test set with a randomly selected, non-repeating patient, labeled as R from the original training/validation set. Subsequently, the algorithm was retrained using the optimal hyperparameters obtained during the previous validation phase.

It’s important to note that for each fold, the original test set patient was integrated into the training set, and vice versa. This patient’s data was used alongside the ground-truth contours provided by the respective expert associated with the test set. For example as shown in Table 6, in the first fold, the contours created by expert 1 for patient 1 were utilized, and that patient was replaced with R1. In the sixth fold, the contours produced by expert 2 for patient 1 were considered, and that patient was replaced with R6. This entire process involved conducting a 5-fold repeated cross-validation for each expert, resulting in the evaluation of the algorithm on 5 independent and previously unseen test sets, along with the inclusion of 10 non-repeating random patients from the training/validation set, totaling 15 patients assessed in total.

**Table 6:** The generated cross-validation folds or repeated 5-fold cross-validation per expert, includes the patients in the training set (comprising the original Training (T) and Validation (V) sets) and the test sets. The first 5 folds (1-5) use the contours from expert 1, and the next 5 folds (6-10) use the contours from expert 2

Fold	Set type	Test set	Expert
	T : Training	Patient #	Ground
	V : Validation		Truths
1	T , V + 1	(R1,2,3,4,5)	Expert 1
2	T , V + 2	(1,R2,3,4,5)	Expert 1
3	T , V + 3	(1,2,R3,4,5)	Expert 1
4	T , V + 4	(1,2,3,R4,5)	Expert 1
5	T , V + 5	(1,2,3,4,R5)	Expert 1
6	T , V + 1	(R6,2,3,4,5)	Expert 2
7	T , V + 2	(1,R7,3,4,5)	Expert 2
8	T , V + 3	(1,2,R8,4,5)	Expert 2
9	T , V + 4	(1,2,3,R9,5)	Expert 2
10	T , V + 5	(1,2,3,4,R10)	Expert 2

The network was trained on a single Nvidia Titan RTX GPU with 24 GB of memory installed on a workstation running Linux with 128GB of memory. The GPU workstation used an Intel i9-9820X CPU with 20 cores running at 3.30 GHz (Lambda Labs, San Francisco, CA, USA).

### Evaluations

The trained models used a frame-based segmentation labelling approach and produced frame-level predictions, which were evaluated against the frame-based segmentation ground truths provided by the 2 independent experts using the DSC [27] and OVC [28] metrics. The same metrics were used to evaluate the ground-truth contours’ variability between the two experts (AW, MS) (i.e interobserver study) using the experts’ contours generated for the 5 patients belonging to the test set.

The DSC score measures the intersection between the two segmentations as the ratio between the number of pixels intersecting the two segmentations (multiplied by 2) and the sum of the total number of pixels included in each segmentation. Thus, this metric ranges from 0 to 1, where ‘1’ represents a perfect match between the two segmentations.

The OVC score measures the overlap between 2 finite sets or 2 segmentations, by showing how much (value between 0 and 1) of the smaller segmentation is enclosed or contained within the larger segmentation. This metric accounts for and is sensitive to the relative location of the 2 finite sets (segmentations) while the DSC is not. A small DSC score can result from the segmentations barely intersecting/overlapping one another or from one segmentation being within the other when the size difference between them is significant. The OVC serves as a supplementary evaluation metric when combined with the DSC score and provides useful information in our study when comparing segmentation masks of differing sizes.

## Results

The worst to best performing average DSC scores for the test set are shown per fold (Table 7), per video (Table 8), and per patient (Table 9). Where the training of the algorithm (on an equal number of expert 1 and expert 2 ground truths) has demonstrated performance on par with or higher than the experts’ evaluation (i.e., interobserver study).

**Table 7:** The average DSC scores per fold (F) between the algorithm’s predicted segmentations and each expert’s segmentation labels, ordered from worst to best DSC

F	Algorithm / Expert 1	F	Algorithm / Expert 2
9	0.662 +/- 0.15	8	0.656 +/- 0.14
10	0.689 +/- 0.13	10	0.669 +/- 0.14
4	0.690 +/- 0.12	5	0.692 +/- 0.11
6	0.692 +/- 0.16	2	0.693 +/- 0.13
7	0.698 +/- 0.11	3	0.695 +/- 0.10
3	0.707 +/- 0.10	7	0.710 +/- 0.12
2	0.721 +/- 0.11	4	0.712 +/- 0.15
1	0.724 +/- 0.12	6	0.747 +/- 0.10
8	0.725 +/- 0.07	1	0.757 +/- 0.10
5	0.727 +/- 0.12	9	0.768 +/- 0.09

**Table 8:** The average DSC scores per video of the test patients (ranked worst to best) between the algorithm’s predicted segmentations and each expert ground truth segmentation labels. Each patient (P) in the test set included 2 videos labelled as a and b

P	Expert 1 / Expert 2	P	Algorithm / Expert 1	P	Algorithm / Expert 2
4b	0.312 +/- 0.10	4b	0.564 +/- 0.02	4b	0.569 +/- 0.02
1a	0.449 +/- 0.06	2b	0.604 +/- 0.03	1a	0.573 +/- 0.04
2b	0.550 +/- 0.05	4a	0.630 +/- 0.03	1b	0.574 +/- 0.04
1b	0.563 +/- 0.08	1b	0.632 +/- 0.06	4a	0.601 +/- 0.03
5b	0.593 +/- 0.05	1a	0.649 +/- 0.06	2b	0.684 +/- 0.03
5a	0.665 +/- 0.10	5a	0.719 +/- 0.06	5b	0.776 +/- 0.02
4a	0.671 +/- 0.06	5b	0.721 +/- 0.05	5a	0.820 +/- 0.01
3b	0.743 +/- 0.04	2a	0.830 +/- 0.02	3b	0.830 +/- 0.02
2a	0.762 +/- 0.05	3b	0.860 +/- 0.03	3a	0.853 +/- 0.01
3a	0.772 +/- 0.02	3a	0.898 +/- 0.02	2a	0.864 +/- 0.02

**Table 9:** Average DSC scores for the 5 patients (P) in the test set (ranked from worst to best) are shown for the interobserver study (expert 1 / expert 2) and the algorithms performance computation (algorithm / expert 1 and algorithm / expert 2) against each expert

P	Expert 1 / Expert 2	P	Algorithm / Expert 1	P	Algorithm / Expert 2
4	0.491 +/- 0.20	4	0.586 +/- 0.04	1	0.573 +/- 0.04
1	0.506 +/- 0.09	1	0.627 +/- 0.06	4	0.581 +/- 0.03
5	0.629 +/- 0.09	2	0.714 +/- 0.12	2	0.765 +/- 0.09
2	0.656 +/- 0.12	5	0.714 +/- 0.05	5	0.783 +/- 0.03
3	0.758 +/- 0.03	3	0.870 +/- 0.03	3	0.834 +/- 0.02

**Fig. 4:**
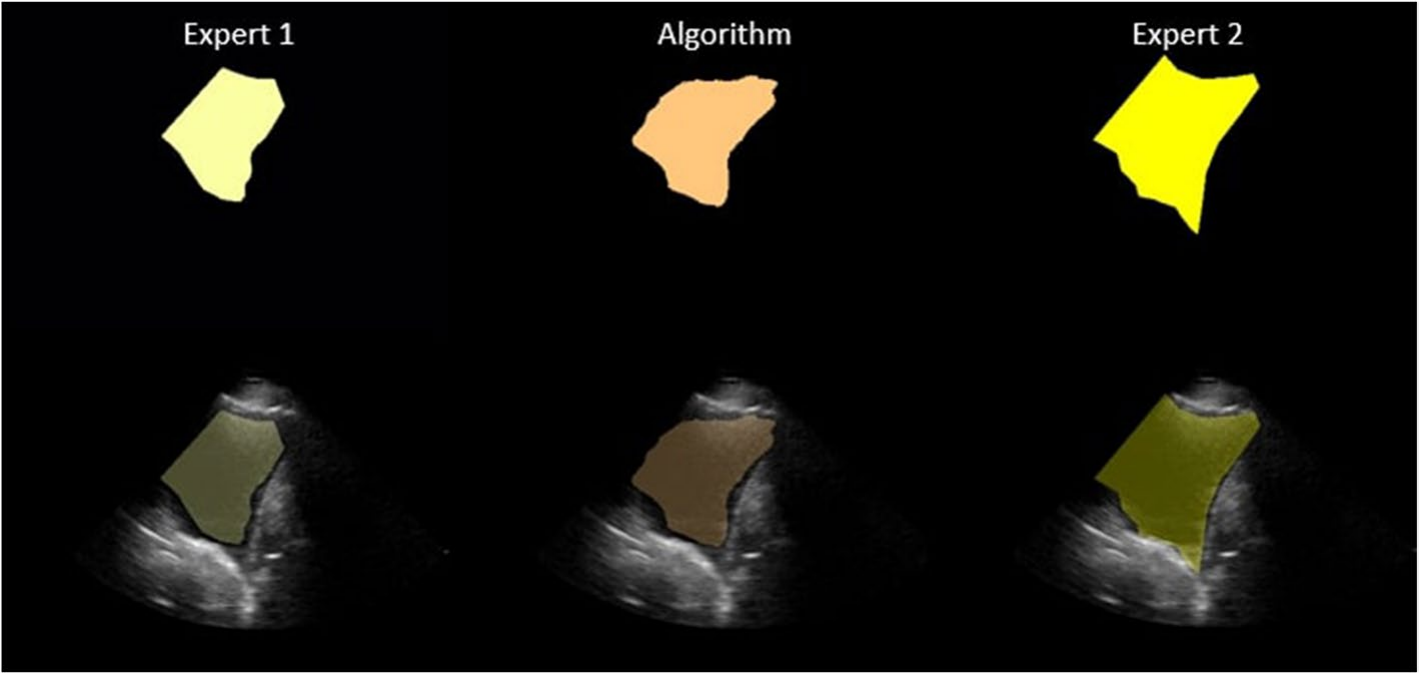
An example of a LUS image and corresponding segmentations generated by expert 1, expert 2, and the algorithm (from left to right) respectively. On the bottom row, the LUS image and the segmentation are overlayed; on the top row, only segmentations are shown

**Fig. 5:**
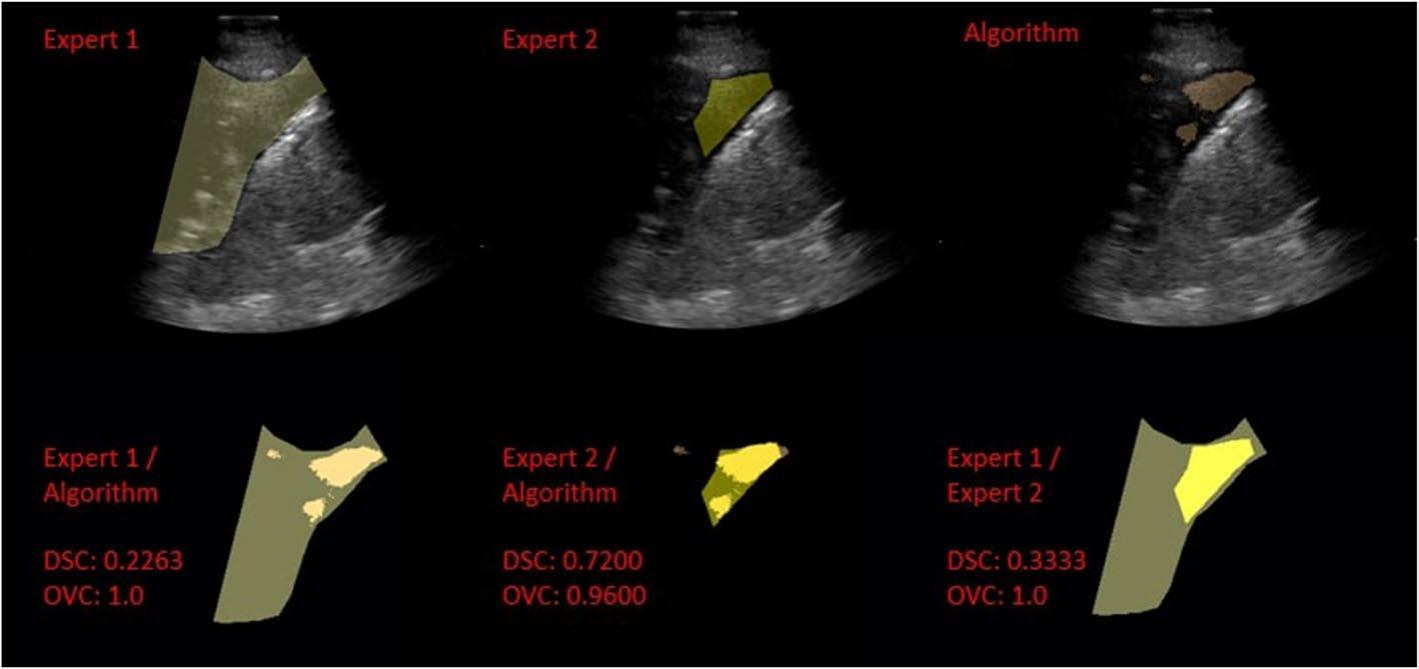
An example of a LUS image and corresponding segmentations generated by expert 1, expert 2 and the algorithm (from left to right), respectively, extracted from the worst performing video in terms of DSC (i.e., 4b from Table 8). Overlays of LUS image and segmentation are shown in the top row. The bottom row shows the DSC and OVC scores calculated between algorithm / expert 1, algorithm / expert 2, and expert 1 / expert 2 segmentations

Figure 5 shows a representative example where there was a high overlap (OVC) between the segmentations generated, in contrast to a low DSC. The high OVC indicates that both experts agree on a common area where PE is located, whilst there is a mismatch in the size of PE (and, thus, low DSC). This is further shown in Table 11 where video 4b is closer to the best average OVC score. When the segmentations are closer in size as in Fig. 4 and are contained within one another, the OVC and DSC calculation are similar (high DSC and OVC).

**Table 10:** The OVC scores for the cross-validation results shown per fold (F) from worst to best OVC

F	Algorithm / Expert 1	F	Algorithm / Expert 2
8	0.874 +/- 0.11	4	0.873 +/- 0.10
10	0.877 +/- 0.12	9	0.891 +/- 0.07
5	0.895 +/- 0.12	7	0.902 +/- 0.06
3	0.898 +/- 0.11	10	0.909 +/- 0.10
2	0.908 +/- 0.11	2	0.916 +/- 0.05
6	0.916 +/- 0.12	5	0.920 +/- 0.06
1	0.924 +/- 0.11	1	0.924 +/- 0.05
4	0.932 +/- 0.10	6	0.930 +/- 0.07
7	0.936 +/- 0.09	3	0.931 +/- 0.05
9	0.939 +/- 0.10	8	0.936 +/- 0.03

In Table 10, the OVC scores are shown from worst to best between the algorithm and each expert (algorithm / expert 1 and algorithm / expert 2).

**Table 11:** The worst to the best average OVC scores shown for the 5 patients (P) and their respective videos (labelled a and b) in the test set

P	Expert 1 / Expert 2	P	Algorithm / Expert 1	P	Algorithm / Expert 2
2b	0.624 +/- 0.10	2b	0.713 +/- 0.02	1a	0.822 +/- 0.09
4a	0.950 +/- 0.09	4a	0.743 +/- 0.02	4a	0.824 +/- 0.04
1b	0.989 +/- 0.03	1b	0.798 +/- 0.06	1b	0.862 +/- 0.09
5a	0.997 +/- 0.01	1a	0.933 +/- 0.04	5a	0.911 +/- 0.02
3a	0.998 +/- 0.01	5b	0.975 +/- 0.01	5b	0.924 +/- 0.04
1a	0.998 +/- 0.01	2a	0.982 +/- 0.01	2b	0.932 +/- 0.02
4b	0.998 +/- 0.01	3b	0.988 +/- 0.01	2a	0.952 +/- 0.03
2a	0.999 +/- 0.01	5a	0.988 +/- 0.01	4b	0.955 +/- 0.01
3b	0.999 +/- 0.01	4b	0.990 +/- 0.01	3b	0.962 +/- 0.02
5b	0.999 +/- 0.01	3a	0.991 +/- 0.01	3a	0.989 +/- 0.01

In Table 11, the OVC scores are shown for the interobserver study (expert 1 / expert 2) and the algorithm’s performance against each expert (algorithm / expert 1 and algorithm / expert 2).

**Table 12:** The average OVC scores for the test patients (P) shown from the worst to the best patient

P	Expert 1 / Expert 2	P	Algorithm / Expert 1	P	Algorithm / Expert 2
2	0.812 +/- 0.20	2	0.847 +/- 0.14	1	0.842 +/- 0.09
4	0.974 +/- 0.07	4	0.866 +/- 0.12	4	0.889 +/- 0.07
1	0.994 +/- 0.02	1	0.866 +/- 0.09	5	0.917 +/- 0.03
5	0.998 +/- 0.01	5	0.981 +/- 0.01	2	0.942 +/- 0.03
3	0.999 +/- 0.01	3	0.989 +/- 0.01	3	0.975 +/- 0.02

The algorithm’s OVC score performance per patient and per video in Tables 11 and 12 vs the interobserver study (expert 1 / expert 2) was on par overall and performed higher when compared to the worst performing interobserver patient study (patient 2).

**Fig. 6:**
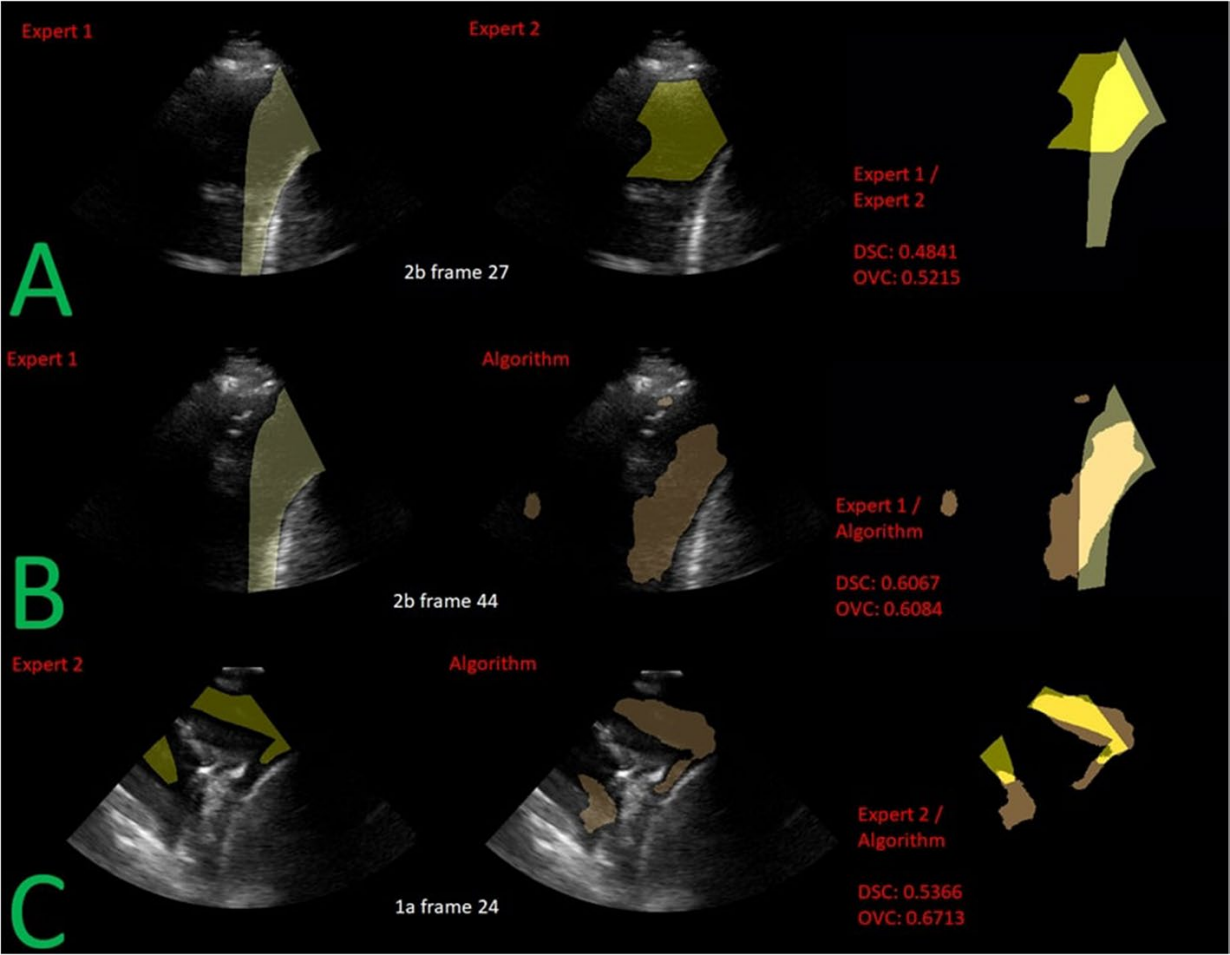
An example of the corresponding segmentations from expert 1, expert 2 and the algorithm’s prediction shown overlayed over the LUS frame of the worst performing DSC video (i.e., 2b and 1a from Table 11). Each row (**A**, **B**, **C**) shows the worst performing LUS frame of the worst performing video for the interobserver study, algorithm / expert 1, and algorithm / expert 2 respectively

In Fig. 6, the segmentations overlap even for the worst performing OVC cases. In image A, the low OVC score indicates that the low DSC score is due to a slight misalignment, in contrast to Fig. 5 where the segmentations are enclosed within each other, resulting in a high OVC score. OVC paired with DSC provides more information on the segmentation overlaps, whether it is an enclosure as in Fig. 5 or relatively minor misalignment as in Fig. 6 (images A and B).

## Discussion

Our study details a DL algorithm that achieved an average DSC between 0.57 and 0.89 at PE segmentation on LUS images. We demonstrated that its performance was comparable to the experts’ interobserver variability, which ranged between 0.31 and 0.77 DSC.

Interestingly, Morilhat et al. [12] proposed a DL-based PE segmentation model that achieved a median DSC of 0.74 and 0.82 on two datasets acquired using a linear and curvilinear transducer, respectively. Our study differs from Morilhat et al. in several aspects. Firstly, our LUS dataset consisted of images acquired using a phased-array transducer. Secondly, most of our training set consisted of images from the posterior lower zone, following the iLungScan protocol [17]. The anatomical landmarks in the posterior lower scanning zone usually include the visualization of the diaphragm. The resulting images may differ from those taken at the BLUE-protocol’s PLAPS-point [29] and the subcostal view. Thirdly, it’s worth noting that most of the pleural effusions in our study exhibited a transudative (anechoic) appearance, primarily because our patients suffered from acute exacerbation of heart failure, and the effusions were located in the lower posterior regions of the lung. This differs from the characteristics of tuberculous pleural effusions, which typically present as exudative (echogenic) in nature. However, it’s important to clarify that the definitive differentiation between exudative and transudative cases is typically based on clinical criteria and laboratory analysis, such as through needle aspiration, rather than relying solely on ultrasound or other imaging modalities.

Lastly The clinical dataset employed in this study is, admittedly, relatively small in scale. However, despite this constraint, the algorithm showcased its effectiveness in this proof-of-concept study by outperforming the interobserver study. It effectively identified and localized PE within the LUS images.

In the future, it will be particularly interesting to compare this algorithm and Morilhat et al.’s algorithm’s performance on both the tuberculous PE dataset and the internal medicine dataset used in this study.

Regarding other common lung pathologies, a comparison could be made to works from Roy et al. [14]. They recently demonstrated a binary DSC of 0.75 at segmenting COVID-19 associated pathologies, including pleural line abnormalities and consolidations. However, caution should be taken when making the comparison, given the heterogeneity in artifact appearance between PE and the features noted in the COVID-19 study. It is to be noted that our algorithm has been trained to address a single pathology, although concurrent pathologies such as lung collapse/consolidations and interstitial syndrome are common in patients with PE.

For interobserver variability between the two LUS experts, we reported the lowest average DSC of 0.31 in video 4b (Fig. 5). Video 4b was obtained from the posterior lower zone. To the right of the image were the diaphragm and liver. To the left and in the lower edges of the image, there was echogenicity suggesting the presence of either a collapsed lung, visceral pleura, or complex effusion. Our investigation into the discrepancies of the contours generated by the two experts determined that Expert 1’s ground truths likely accounted for images from the preceding and subsequent frames to help determine the extent of the effusion, respectively. In contrast, Expert 2’s ground truths were probably restricted to an anechoic region of the highest confidence. In retrospect, the LUS experts likely overestimated and underestimated the extent of the effusion. As expected, since the DL algorithm was trained using the annotations of both experts, it was able to produce a segmentation that was a ‘compromise’ between the two experts. In fact, the automated segmentation primarily included regions where the two experts agreed (OVC > 0.95 for the worst DSC video). Most notably, the segmentation in video 4b excluded less-certain areas (where the boundaries of PE were unclear) that may be unsafe for the introduction of a needle or catheter during procedures such as ultrasound-guided thoracentesis. Given the significant consequences of injuring the lung tissue or diaphragm, we believe our algorithm is also a step in the right direction for safeguarding patients with PE against iatrogenic complications. The OVC alongside the DSC can be used to determine safe catheter placement during thoracentesis needle insertion.

The highest interobserver DSC reported was 0.77 (e.g Fig. 4), similar to that observed by Morilhat et al. [12]. Other common measures of interobserver variability are Cohen’s kappa and Fleiss’ kappa. In a recent COVID-19 lung study, Kumar et al. [30] reported a moderate agreement in the presence (kappa = 0.49) and size of PE (kappa = 0.47). A similar finding was also reported in the paediatric population [31] (kappa = 0.44). It is evident that large variation exists in determining the presence of PE, and agreement remains challenging.

This study has several limitations. First, the study was conducted using LUS images from patients with cardiopulmonary complaints in a single tertiary center. The images were obtained by a single operator using the phased-array transducer from a single manufacturer. The generalizability of the DL algorithm to other clinical and image acquisition settings is to be determined. Second, most of the videos were from the posterior lower scanning zones. A larger sample size that includes effusions detected in various scanning zones, such as the posterior upper zone in a supine patient or anterior zone in a prone patient, would be beneficial. However, it’s worth noting that these factors don’t inherently limit the algorithm’s performance, as it can identify and localize imaging patterns associated with PE. The variation in imaging/scanning protocols primarily influences the location of PE within the scan, rather than the algorithm’s capability to identify and localize these patterns. Where PE incidents are primarily prevalent in unilateral cases [32, 33]. However, they can also occur bilaterally, with most cases being right-sided PE [34]. Notably, right-sided PE cases tend to feature larger effusions compared to the left side [35]. The most common cases of PE are attributed to heart failure [33]. Previous research findings indicate that the location of PE is not an unusual finding, particularly in cases related to heart failure [36]. This study concentrated on an imaging protocol [17] where most cases of PE were located in the lower lung regions, and our algorithm was trained specifically for these scenarios. In the future, we intend to broaden our dataset to ensure the algorithm’s effectiveness in identifying this pathology in various lung regions.

Third, the LUS images were reviewed by two LUS experts retrospectively without complete knowledge of the clinical context. This differs from a typical POCUS routine, where the operator acquires and interprets the images to address a specific clinical question. The agreement between experts may improve if the experts acquire and interpret the images in real-time.

The DSC score metric is the standard evaluation metric for segmentation tasks in machine learning (ML). The challenge with the DSC score arises from the need for the size of ground truth and algorithm-predicted segmentations to be the same or relatively similar in size. PE size in LUS images has shown from our study and others [30] that the experts often disagree on the exact size and boundary of PE effusion and that the DSC, at least for PE segmentation, is not sufficient on its own.

Future work may consider the clinical utility of the algorithm output against patient safety. For example, how often the algorithm-outlined PE segmentations are safe for thoracentesis needle insertion. This trained algorithm will also benefit from being tested in other patient cohorts where PE may appear differently (for instance, complex PE with fibrotic materials within the pleural space) to increase the dataset size that could be used for training. Ultimately, a comprehensive tool for PE will likely require consideration of the pretest probability of the pleural effusion and assess the clinical significance of the effusion against the patient’s demographic information and clinical history.

Moreover, considering the enhancement of Pleural Effusion (PE) segmentation for improved volume estimation during thoracentesis, the natural progression involves incorporating spatial information from volumetric Lung Ultrasound (LUS) images into the estimation process. To advance the automatic PE segmentation algorithm, exploration can extend to its application to three-dimensional LUS images. Additionally, various Machine Learning (ML) approaches such as transformers, transfer learning, reinforcement learning, and unsupervised learning can be harnessed to enhance LUS segmentation accuracy in future iterations.

As a preliminary step or alternative approach, consideration can be given to a 2D image fusion technique inspired by Ziyan Zhang et al.’s work [37]. This technique involves using Gaussian pyramids to seamlessly combine 2D data, presenting an intriguing avenue for exploration. Applying this method to the existing dataset could lead to the transformation of a 2D LUS video into a single, larger 2D image. This consolidated image offers a comprehensive view of the scanning region, effectively showcasing the contribution of each frame. Such an approach could prove invaluable for conducting a more detailed and comprehensive analysis of the lung’s surface during LUS video assessments.

## Conclusion

We proposed an automatic PE segmentation of LUS videos using a DL approach. When compared to experts, we demonstrated the algorithm’s capability of segmenting PE on LUS with an average DSC between 0.57 and 0.89. We showed a DSC between 0.31 and 0.77 between the two experts, suggesting a significant degree of variability in PE segmentation. We observed that the algorithm avoided segmenting high-risk, high-uncertainty regions such as potential lung tissues on LUS. These early results are promising for the growing field of ML assisted medicine.

Future focus will be on the generalizability of the algorithm in other settings and datasets, addition of LUS experts in the interobserver study, inclusion of an independent expert(s) to review and score algorithms segmentation based on safety of ‘needle placement’ during PE drainage procedures (thoracentesis), testing of other DL approaches (transformers, transfer learning, transformers, etc) for PE segmentation, and consideration of volumetric estimation using automated segmentation on three-dimensional ultrasound images.

## Data Availability

The datasets used and/or analysed during the current study are available from the corresponding author(s) on reasonable request.
